# Restoring Sensorimotor Function Through Neuromodulation After Spinal Cord Injury: Progress and Remaining Challenges

**DOI:** 10.3389/fnins.2021.749465

**Published:** 2021-10-14

**Authors:** Hui Zhang, Yaping Liu, Kai Zhou, Wei Wei, Yaobo Liu

**Affiliations:** Jiangsu Key Laboratory of Neuropsychiatric Diseases and Institute of Neuroscience, Soochow University, Suzhou, China

**Keywords:** spinal cord injury, neuromodulation, optogenetics, electrical stimulation modulation, sensorimotor function, neural circuits

## Abstract

Spinal cord injury (SCI) is a major disability that results in motor and sensory impairment and extensive complications for the affected individuals which not only affect the quality of life of the patients but also result in a heavy burden for their families and the health care system. Although there are few clinically effective treatments for SCI, research over the past few decades has resulted in several novel treatment strategies which are related to neuromodulation. Neuromodulation—the use of neuromodulators, electrical stimulation or optogenetics to modulate neuronal activity—can substantially promote the recovery of sensorimotor function after SCI. Recent studies have shown that neuromodulation, in combination with other technologies, can allow paralyzed patients to carry out intentional, controlled movement, and promote sensory recovery. Although such treatments hold promise for completely overcoming SCI, the mechanisms by which neuromodulation has this effect have been difficult to determine. Here we review recent progress relative to electrical neuromodulation and optogenetics neuromodulation. We also examine potential mechanisms by which these methods may restore sensorimotor function. We then highlight the strengths of these approaches and remaining challenges with respect to its application.

## Introduction

The most obvious consequence of spinal cord injury (SCI) is paralysis, which leaves the patient with partial or complete loss of sensation and movement. SCI also affects many bodily functions, such as bladder, bowel, respiratory, cardiovascular, and sexual functions (Cowan et al., 2020). Between 10 and 80 million people suffer from SCI each year throughout the world (Chari et al., 2017; Schwab et al., 2018). Current treatment for acute SCI is limited to surgical decompression and intravenous high-dose methylprednisolone, but recent clinical studies have found that high doses of methylprednisolone significantly increase the incidence of many complications (such as pneumonia, bedsores, and blood clots) (Pizzolato et al., 2021). At present, SCI cannot be cured completely, and the recovery of meaningful voluntary motor control after complete injury is very limited (Minassian et al., 2016).

SCI leads to the loss of nerve and blood vessel cells and destroys the normal connection between spinal cord neural circuits and the cerebral cortex, resulting in the destruction of neuromuscular communication and thus permanent neurological dysfunction. Recovery of neural function depends on the enhancement of neural plasticity. Such enhanced neural plasticity can promote the germination and regeneration of damaged axons, increase the strength of residual connections, promote the formation of new correct connections, and neural circuits and ultimately promote the recovery of sensory and motor functions. Neuroplasticity can be achieved through four main biological properties: the internal signaling of neurons, the external environment of neurons, the reconnection of the severed spinal cord *via* neural stem cell transplantation, and the modulation of neuronal activity (Pizzolato et al., 2021).

Modulation of neuronal activity is mainly achieved by neuromodulation, including pharmacological modulation, electrical modulation, and optogenetics modulation. Pharmacological modulation acts mainly through neuromodulators, such as small molecule transmitters, biogenic amines, neuropeptides, and others, which target ion channels and synapses and alter the dynamics of neural circuits. Pharmacological modulation has been reviewed previously (Marder, 2012; Nadim and Bucher, 2014; Sillar et al., 2014; Hutson and Di Giovanni, 2019), and will not be covered here.

Electrical modulation is mainly achieved by brain stimulation, spinal cord stimulation, peripheral stimulation, and a brain-machine interface (BMI). Electrical modulation therapy for SCI has developed rapidly in recent years and has achieved impressive results. Cortical stimulation, which includes transcranial direct current stimulation (tDCS), transcranial magnetic stimulation (TMS) and direct motor cortex stimulation (MCS), and deep brain stimulation(DBS) are the main forms of brain stimulation. Spinal cord stimulation mainly includes epidural electrical stimulation (EES) and transcutaneous spinal cord stimulation (tcSCS). Functional electrical stimulation (FES), which is the main form of peripheral stimulation, is currently the most well-developed form of neuromodulation for SCI. BMI leads to recovery of motor ability by completely bypassing the injured interface. The development of electrical stimulation modulation has been largely limited by the lagging development of neuromodulation devices especially with respect to their durability, availability, price, and operation and the limited understanding of electrical modulation mechanisms. Optogenetics modulation (Song et al., 2019) mainly uses optogenetics technology to regulate changes in the transmembrane protein configuration of related neurons and to change the neuronal membrane potential, thus completing the regulation of the neural network (Xiao et al., 2015). The continuous development of optogenetics technology is expected to overcome the limitation of electrical stimulation in promoting sensorimotor function recovery after SCI. The combination of multiple therapeutic approaches may be one of the main ways to achieve precise and effective treatment. In this review, we will introduce the recent progress, potential mechanisms, and future challenges of electrical modulation and optogenetics modulation in the treatment of SCI.

## Electrical Stimulation Neuromodulation

### Brain Stimulation

#### Cortex Stimulation

There are three main forms of cortical stimulation: tDCS, TMS, and MCS. tDCS is a non-invasive neuromodulation method that works through two or more scalp electrodes. Clinically, tDCS is often used as a research tool to explore the role of different brain regions and is used as a treatment for psychiatric disorders such as obsessive-compulsive disorder, depression, schizophrenia, and addiction. When tDCS is used to treat SCI, the most common protocol is to combine it with motor training to promote activity-dependent plasticity. For example, Gomes-Osman and Field-Fote (2015) showed that a meaningful improvement in hand function among SCI patients was observed when tDCS was combined with the practicing of hand functional tasks. The priming effect of tDCS is time dependent (Sriraman et al., 2014), and anodic tDCS can improve motor performance more than cathodic tDCS (Machado et al., 2019), which may be the key regulatory condition determining the therapeutic effect of tDCS. In addition, Murray et al. (2015) demonstrated that stimulation of 1 or 2 mA during tDCS improved sensory perception. Unfortunately, there are no reports that tDCS promotes recovery of lower limb motor ability after SCI, with only one pilot study (National Clinical Trial, NCT03237234) having been carried out (Raithatha et al., 2016), the results of which may inform the efficacy of tDCS in subsequent clinical trials. However, tDCS is still very popular in clinical practice due to its non-invasive nature, and low cost, as well as its good therapeutic effect on a variety of neurological diseases and recovery of upper-limb function.

**Figure 1:**
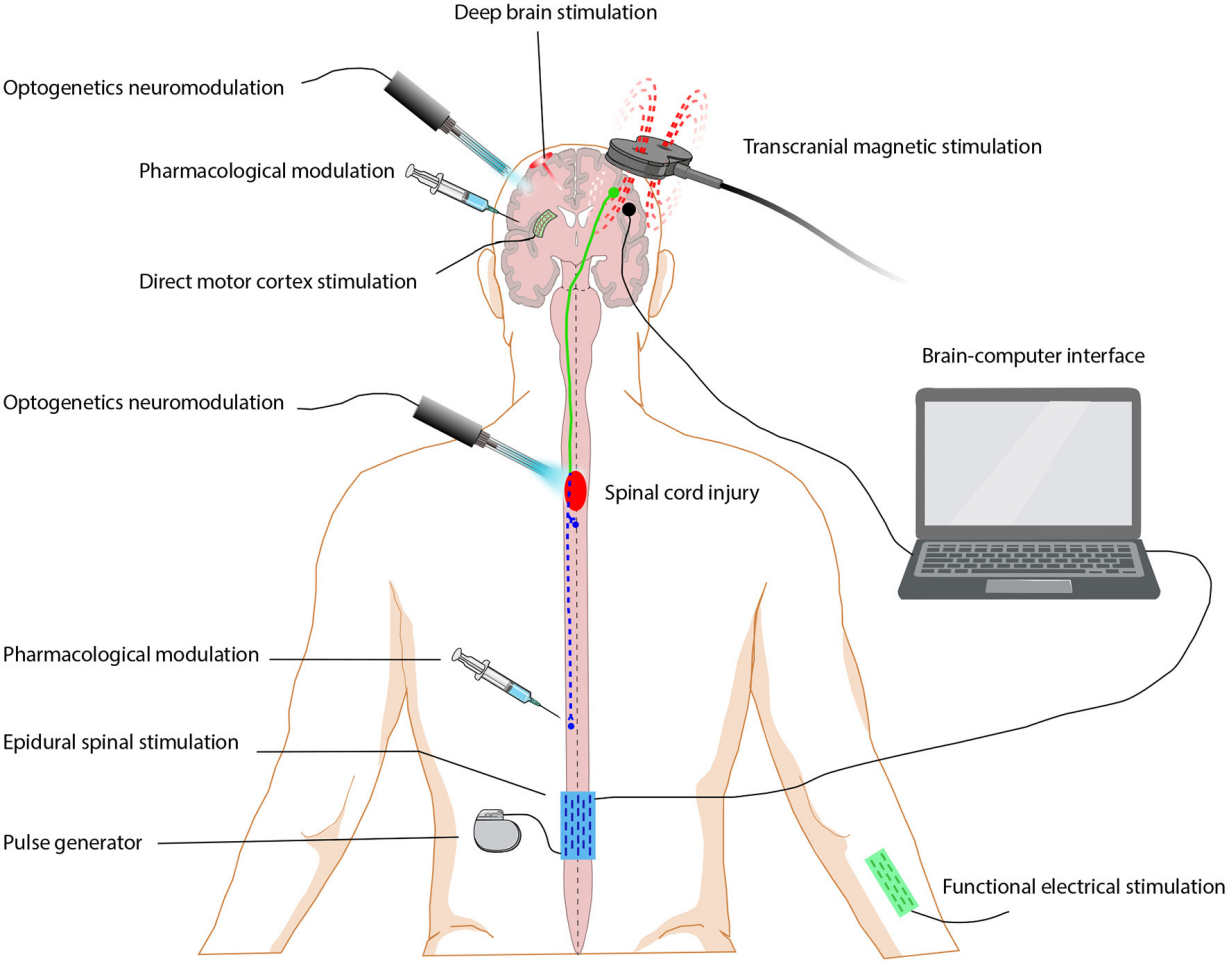
Neuromodulation approaches for restoring function after spinal cord injury. Neuromodulation includes pharmacological modulation, electrical modulation and optogenetics modulation. Electrical modulation approaches are grouped by the stimulation location, including brain stimulation (deep brain stimulation, direct motor cortex stimulation), spinal cord stimulation (epidural electrical stimulation), peripheral stimulation (functional electrical stimulation) and brain-machine interface.

TMS is a safe, non-invasive brain stimulation technique that can also be used to treat psychiatric disorders such as addiction and depression and movement disorders. The magnetic stimulator consists of a strong magnetic field that can discharge a large current through an induction coil placed on the scalp (see Figure 1). A single TMS pulse in the primary motor cortex is paired with electrical stimulation of the peripheral nerves, such that corticospinal synaptic transmission is enhanced and plasticity of the residual corticospinal projection is induced (Christiansen and Perez, 2018). There is growing evidence that exercise combined with non-invasive stimulation targeting spinal synapses further promotes functional recovery. The amplitude of corticospinal responses elicited by TMS and the magnitude of maximal voluntary contractions in targeted muscles increased on average by 40–50% after paired corticospinal–motor neuronal stimulation combined with exercise in one study (Jo and Perez, 2020). As behavioral and physiological effects were preserved 6 months after the intervention, this suggests that the stimulation helped to maintain exercise gains. TMS combined with exercise can also benefit the recovery of lower-limb motor function. High-frequency TMS combined with gait training improved the lower extremity motor score and gait velocity in individuals with SCI who were able to walk over ground (Kumru et al., 2016). With a greater understanding of its mechanism of action and improvements in the equipment used to deliver it, TMS may play an important role in clinical treatments (Alexeeva and Calancie, 2016).

MCS with extradural electrodes promotes skilled forelimb function recovery after SCI in rodents, which is attributed to compensatory conjunctive germination of retained corticospinal tract axons (Martin, 2016). A combined spinal-motor cortex neuromodulation approach has been shown to promote the partial recovery of skilled motor behavior in SCI adult rats (Zareen et al., 2017).

Cortical stimulation—either non-invasive stimulation *via* tDCS and TMS or invasive stimulation *via* MCS—activates the corticospinal tract, resulting in immediate adaptive plastic changes or inducing motor output (Chari et al., 2017). Most of these methods can be combined with motor training or spinal cord and peripheral electrical stimulation to achieve the best training effect.

#### Deep Brain Stimulation

DBS is commonly used to treat conditions such as Parkinson's disease, dystonia, and essential tremor. In recent years, studies have found that DBS can also play a role in motor function recovery after SCI treatment. DBS has been experimentally applied to the subcortical motor regions of rats and has led to improvements in hindlimb function in both acute and chronic injuries (Hentall and Gonzalez, 2012; Bachmann et al., 2013). Excitatory DBS of the mesencephalic locomotor region significantly improved hindlimb function in rats with incomplete SCI and even promoted step-like movement after SCI paralysis (Bachmann et al., 2013). One study has shown the synergistic effect of EES and DBS on exercise in a severe SCI contusion rat model (Bonizzato et al., 2021). This approach promoted voluntary movement and authors associated DBS with walking intention, achieving autonomous movement, and reducing the stress response. Clinical improvement of gait in patients with incomplete SCI who were treated with DBS (NCT03053791) has shown that stimulation of the mesencephalic locomotor region induces forced movement and can transcend any volitional control (Mahlknecht et al., 2015). DBS-mediated brainstem repair is simple and comprehensive, although our lack of understanding concerning its mechanism of action currently limits its further application.

### Spinal Cord Stimulation

#### Transcutaneous Spinal Cord Stimulation

tcSCS, in which electrodes are placed on the skin above the spine to stimulate the dorsal roots and activate the motor circuit, is a non-invasive treatment for SCI. This method improves upper-limb function at the C3 level in patients with chronic incomplete SCI (Inanici et al., 2018). In another study, six subjects with motor complete injury were treated with tcSCS and buspirone, which is an important and prevalent neurotransmitter system for locomotor function. As a result, mean hand strength increased by >300%, and a corresponding clinically significant improvement was observed in upper extremity motor scores and the action research arm test. Some functional improvements persisted for an extended period after the study interventions were discontinued (Freyvert et al., 2018). tcSCS can increase active motor responses in the upper and lower extremities and trunk stability and can improve function and quality of life in patients with SCI. Although tcSCS is non-invasive, inexpensive and commercially accessible, it lacks the temporal and spatial precision of EES, which can activate specific muscle groups in a step cycle. In addition, the absence of clinical trials with appropriate control groups, has thus far made it impossible to know whether the motor response obtained through spinal cord stimulation is superior to the effects of other interventions used in therapy (Megía García et al., 2020).

#### Epidural Electrical Stimulation

EES, in which electrodes are surgically implanted on the dorsal surface of the spinal cord (see Figure 1), was initially used to treat chronic pain. EES also plays an important role in the recovery of motor and sensory function after SCI. van den Brand et al. (2012) designed electrochemical neural prosthetics and robotic posture interfaces to restore autonomous motor control in paralyzed rats. Dynamic task-specific training in the presence of EES, has been used to enable patients to walk independently across the ground while maintaining hip balance. This is the first report of the realization of independent walking by task-specific training in the EES environment for people who have completely lost sensorimotor function of lower limbs due to SCI (Gill et al., 2018). After EES and treadmill training in four patients with SCI, all four patients achieved independent standing strength and physical stability, and two of them were able to walk on the ground (Angeli et al., 2018). Another research group introduced neural techniques targeting spinal cord stimulation to achieve optimized real-time neuromodulation, enabling patients with permanent motor deficits or complete paralysis to walk autonomously under spatiotemporal stimulation (Wenger et al., 2014; Wagner et al., 2018). EES has also been found to enhance seated reaching-performance of individuals with chronic SCI (Gill et al., 2020). However, EES usually needs to be combined with frequent rehabilitation training, which is not suitable for a large proportion of SCI patients. To solve this problem, Gorgey et al. (2020) proposed that combined exoskeletal-assisted walking for SCI patients may be a feasible rehabilitation method, and preliminary positive results were obtained in patients with C7 complete SCI.

EES improved not only SCI hindlimb motor function but also forelimb fine motor function after cervical SCI. Alam et al. (2017) found that cervical EES increased the success rate of reaching and grasping in rats with cervical SCI, suggesting that cervical epidural spinal cord stimulation has therapeutic potential for rehabilitation after cervical SCI. Greiner et al. (2021) combined a computational model of the cervical spinal cord with experiments in rhesus monkeys and found that the muscle response to EES was modulated during exercise. There is also clinical evidence, such as a case study of 25 participants with cervical SCI who reported improvements in grip strength and motor scores 1 week after daily epidural spinal cord stimulation (Lu et al., 2016).

In recent years, EES has been found to be helpful for sensory recovery after SCI. A study has shown that EES induced somatosensory perception in four individuals with upper-limb amputations. Because restoring somatosensory feedback in amputations is critical for improving prosthetic control, this has important implications for the development of better prosthetic limbs that rein state certain sensations (Chandrasekaran et al., 2020).

### Peripheral Stimulation

FES is the main form of peripheral stimulation. In FES, low-level electrical pulses are applied to paretic or paralyzed muscles to restore or improve their functional capacity (see Figure 1) (Thompson et al., 2014). In clinical and community settings, one of the most commonly available and well-studied FES exercise modalities is FES-evoked cycling (Crosbie et al., 2014). van der Scheer et al. (2021) summarized FES cycling exercise intervention studies found that FES cycling exercise improved lower-body muscle health (e.g., muscle mass, fiber type composition) in adults with SCI. Dolbow et al. (2012) found that different FES training paradigms produced different responses, with 35-Hz FES stimulation resulting in a greater ability to stand up among patients. This suggests that different therapeutic parameters of FES may directly influence therapeutic outcomes, underscoring the need to decode the specific mechanisms involved. The greatest advantages of FES are its safety and the availability of stimulation devices. FES is one of the few electrical stimulation therapy regimens that can be carried out with a home-based FES lower extremities cycling system (Bouton et al., 2016).

### Brain-Machine Interface

Exciting advances are being made in neuromodulation in the field of BMI (see Figure 1). BMI, which is also referred to as brain-computer interface (BCI), records and decodes motor signals from the motor cortex of a paralyzed human and uses these signals to control artificial limbs, exoskeletons or electrodes to directly influence motor output, thus BMI functions to bypass the injury site (Bouton et al., 2016). When this approach was combined with rehabilitation training, voluntary motor control among patients with SCI was also improved, suggesting that these devices promote activity-dependent neuroplasticity in the brain and/or spinal cord, leading to improvements in neurological function (Alam et al., 2016).

Pfurtscheller et al. (2003) first used the non-invasive electroencephalogram (EEG) to enable quadriplegic patients to control gripping movement through FES activated by modulating sensorimotor rhythm. Subsequently, Hochberg et al. (2012) demonstrated that individuals with long-standing quadriplegia were able to use a neural interface system to move and click a computer cursor, and control a robotic arm to perform reaching. The brain-machine-spinal interface has been used to directly regulate the spinal cord to improve performance on forelimb extension tasks in rhesus monkeys (Zimmermann and Jackson, 2014; Alam et al., 2016) and alleviate gait deficits following SCI in non-human primates (Capogrosso et al., 2016). Compared with continuous spinal cord stimulation, brain-controlled spinal cord regulation enhances long-term motor recovery in rats after severe thoracic contusion (Bonizzato et al., 2018). Eight chronic paraplegics who used immersive virtual reality training over a 12-month period showed significant improvements in both sensory and motor performance. This was the first time that long-term BMI training had been shown to improve neurological function in animals or humans (Donati et al., 2016). In addition, it has been pointed out that the integration of BMI with the sensory cortex will further improve flexibility and fine control (Rosenfeld and Wong, 2017).

Current BCI protocols use either implanted electrodes or non-invasive surface electrodes to extract neural activity information (James et al., 2018). Although electroencephalograms (EEGs) are less invasive than approaches using penetrating microelectrodes, they cannot record the action potential of a single neuron and can measure only the average voltage waveform across a population of thousands of neurons (Rosenfeld and Wong, 2017). The use of penetrating microelectrodes does provide a signal with the highest fidelity but also causes tissue damage and glial growth around the implant site, resulting in signal loss over several months (Rosenfeld and Wong, 2017). The use of electrocorticography (ECoG) arrays may be more appropriate because these arrays offer higher temporal and spatial resolution than EEG electrodes and are less invasive than intracranial microelectrode arrays. However, the biocompatibility of the implanted material, the electrode design, and the minimization of glial growth and electrode corrosion around the electrodes remain key challenges for the further development of BCI (Formento et al., 2018). The development of optical brain imaging technology, optical BCI (OBCI), allows the conversion of brain activity modulation related to motion images into control commands for external devices. Recent experiments have shown that an OBCI device can restore certain upper-limb functions (Soekadar et al., 2021). Trautmann et al. (2021) developed an OBCI driven by dendritic signals in rhesus monkeys and successfully decoded the direction of motion online.

## Optogenetics Neuromodulation

**Figure 2:**
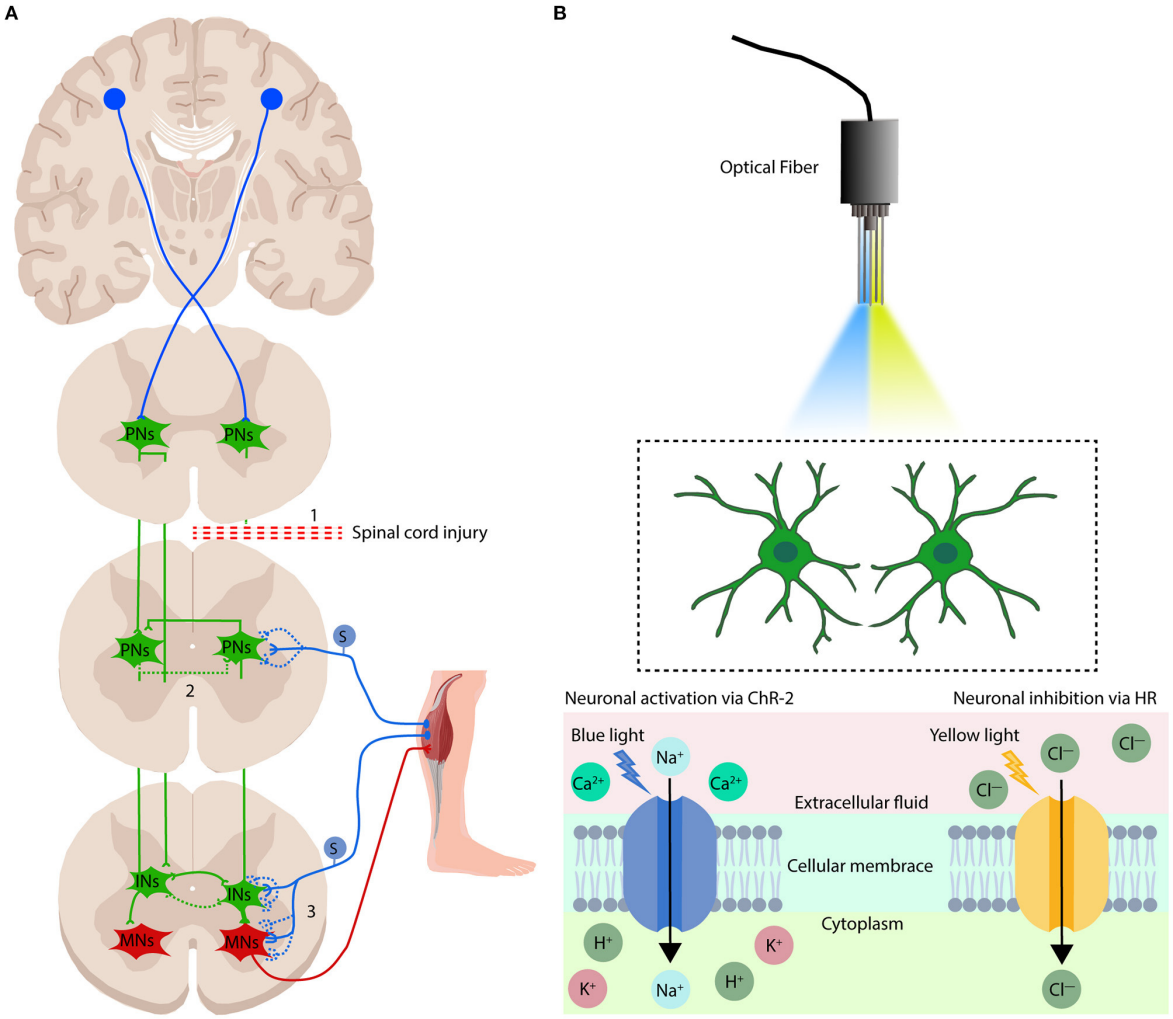
Mechanisms of electrical modulation and optogenetics modulation in the treatment of spinal cord injury. **(A)** There are three potential mechanisms by which electrical stimulation regulation promotes sensorimotor function repair after spinal cord injury: (1) cortical stimulation promotes regeneration of the free ends of the CST; (2) spinal cord stimulation can promote the formation and activation of circuits established by spared PNs that lead to the re-emergence of locomotion and sensation when SCI results in disruption of the flow of motor instructions from the brain and brainstem to the spinal motor circuits; and (3) electrical modulation can recruit proprioceptive afferents, which have been proposed to be the most influential in regaining volitional control of affected muscles, and rebuild the sensorimotor circuits which were disruption when spinal cord injury. PNs, propriospinal neurons; INs, interneurons; MNs, motoneurons. S, sensory neurons. The blue and green dotted lines show the rebuild of neuronal connections after electrical modulation. **(B)** Overview of the optogenetics system. The implanted optical fiber is guided to the target tissue, and the target neuron is activated or inhibited by illumination with blue or yellow light. Specific neurons in the cerebral cortex or spinal cord are simplified into two green neuron patterns in the figure. Blue light (470-nm wavelength) changes the conformation of the transmembrane ion channel protein ChR2, allowing positively charged ions to flow into the cytoplasm, leading to depolarization of neurons. Yellow light (580-nm wavelength) alters the conformation of the transmembrane ion pump protein HR, which allows negatively charged ions to enter the cytoplasm, leading to hyperpolarization of neurons.

Optogenetics relies on the genetic modification of light-sensitive transmembrane proteins (collectively referred to as opsins) and cell-type target genes, which regulates the excitability of neurons with high temporal and spatial precision through the strategy of guiding light through tissues (Zhang et al., 2007; Deisseroth, 2011) (see Figure 2B). These light-sensitive proteins are often used as “gates” to control the activation or inhibition of designated neuronal populations in the central and peripheral nervous system. Aravanis et al. (2007) described a new type of optical neural interface that specifically targets ChR2 to excitatory cells in the cerebral motor cortex by using the CaMKIIα promoter to control motor cortex function and behavioral output, which results in light-based control of the rodent motor system. In addition, in Thy1::ChR2 transgenic mice, light-based control of motor neuron axons results in finely controlled muscle contractions (Llewellyn et al., 2010). These findings have laid the foundation for the widespread application of optogenetics in the field of neuroscience, especially in the regulation of neural circuits with respect to central nervous system damage and repair.

### Advances in SCI-Related Optogenetics Applications

The plasticity and reorganization of the central nervous system are considered to form the basis for functional recovery after SCI. The development of research tools for spinal cord circuits is essential for understanding the loss and recovery of function after SCI-based paralysis. Optogenetics can be used not only to determine the basic mechanism of the spinal cord sensorimotor feedback local circuit after SCI but also to explore the functional integration of axons and spinal cord circuits regenerated after SCI, providing a new strategy for the treatment of SCI.

#### Optogenetics-Based Regulation of Sensorimotor Microcircuits in the Spinal Cord

There is evidence that proprioceptive feedback may provide powerful motor regulation. In particular, excitatory signal input from the periphery can initiate motor output and reset the oscillation period, which is essential for its normal function (Akay et al., 2014; Takeoka et al., 2014). Impaired sensory function leads to a decrease in the tail hopping frequency of free-swimming juvenile zebrafish, indicating that this form of mechanical sensory feedback results in active movement (Bohm et al., 2016). However, the technical challenges of selectively positioning and manipulating sensory pathways make it difficult to explore the contribution of sensory feedback to natural movement. Using the intersectional genetic approach, Gatto et al. (2021) found that multiple excitatory neurons in the superficial dorsal horn (lamina I/II) participated in the protective scratch reflex, whereas the lamina II/III and lamina III/IV induced the paw withdrawal response and balance-related motor correction, respectively, revealing the key principle that spinal soma sensory processing is driven by different spatial complements of excitatory neurons. In addition, light stimulation of cerebrospinal fluid–contacting neurons (CSF-cNs) in zebrafish showed that GABAergic sensory neurons in the spinal cord provide strong inhibitory feedback to the escape circuit during active movement to maintain balance (Hubbard et al., 2016). A recent analysis that used functional anatomy and optogenetics-assisted mapping showed that CSF-cNs can generate synapses on the axons of reticulospinal neurons including Mauthner cells and V2a neurons, and relay their information to the higher-level centers of the hindbrain to stabilize posture and increase movement speed (Wu et al., 2021).

The ability of spinal INs to integrate the spinal cord network and adapt to changing environments has become an important target for treatments aimed at enhancing neuroplasticity and/or promoting repair. They not only play an important role in the regulation of the motor and sensory activities of the undamaged spinal cord but also contribute to plasticity after injury or disease. The development of optical and chemical technologies has gradually improved our ability to distinguish subsets of spinal INs such that we can target specific subpopulation of INs in normal and injured spinal cords, advancing our understanding of spinal INs phenotypes in SCI repair. Capitalizing on this, Alilain et al. (2008) used optogenetics to demonstrate the functional influence of spinal phrenic motor circuitry on diaphragm activity by selectively stimulating transduced motor neurons and INs immediately caudal to a high cervical SCI. Given that sensory afferents are directly received through CPGs under the commissure and proper spinal cord connection, they are projected to the middle and ventral areas of the spinal cord, allowing them to affect the muscles and limbs of multiple joints. Optogenetics regulation of the INs in the spinal cord CPG provides the opportunity to directly observe the connection between the recovery of motor function and the CPG circuit after SCI. Optogenetics can be used to directly target the activation and inactivation of spinal INs and motor neurons in a spasticity model induced by SCI in mice. In one such study, excitatory Ins, and inhibitory INs were recruited into functional circuits by sensory inputs to induce uninterrupted neural activity, revealing the operational logic of spinal cord circuits after injury (Bellardita et al., 2017). Moreover, this spasticity is caused in part by the sensory activation of V3 neurons and the corresponding CPG circuit, which is important in initiating and coordinating motor output after SCI. Optogenetics activation of V3 interneurons in the spinal cord to induce spasms (Lin et al., 2019). Bui et al. (2016) found that motor-related dI3 INs have a similar role. Although dI3 INs are not necessary for normal motor function, they are necessary for the stable recovery of local motor activity after spinal cord transection. Eliminating the glutamatergic output of dI3 INs prevented motor recovery after spinal cord transection, proving that dI3 INs are involved in spinal cord microcircuits that regulate the plasticity of the motor system. Manipulating corticospinal-INs connections by optogenetics, electrophysiological, and transgenic tools have demonstrated that the different corticospinal-INs circuits of corticospinal neurons in the motor cortex and sensory cortex control specific aspects of skilled movement, such as the expression of Chx10 and Vglut3+ (Clovis et al., 2016; Ueno et al., 2018). At present, how to obtain, stimulate and/or enhance the spinal cord network through spinal INs and how to establish the sustainable excitability of damaged and/or denervated circuits are challenges that remain for researchers in this field.

#### Application of Optogenetics Neuromodulation in SCI

More extensive research on promoting axon regeneration in the central nervous system has emphasized the problem of local loop synaptic integration. Since optogenetics technology can directly demonstrate the synaptic integration of regenerating axons and distinguish its function from indirect relay loops and target plasticity, it provides a promising way to explore SCI. Combining the light-induced stimulation of corticospinal tract (CST) sprouted axons with single-cell recordings to evaluate the functional integration ability of CST axons stimulated by sox11 in the cervical spinal cord circuit (Jayaprakash et al., 2016). Furthermore, moderate inhibition of the pericyte-derived glial scar promotes the regeneration of CST axons and improves the recovery of sensorimotor function after SCI, whereas optogenetics stimulation confirmed that the regenerated CST axons and the local spinal cord circuit under the lesion were remodeled (Dias et al., 2018). In addition, gabapentin, a voltage-gated calcium channel α2δ2 subunit inhibitor, promotes the plasticity, and regeneration of the corticospinal structure and the recovery of upper-limb function in adulthood, and the use of optogenetics strategies combined with *in vivo* electrophysiological records proves that these regenerated corticospinal axons are functionally integrated into the spinal cord circuit (Sun et al., 2020). Through optogenetics stimulation of C4 axons, the muscle activation caused by the fine motor circuit can be detected, and this system has been used to verify that administration of interleukin-10 can prevent damage to the pathological electromyography signal of the affected muscle (Chen et al., 2021). In addition, optogenetics targeting of host CST axons that had regenerated following neural stem/progenitor cell (NSPC) grafts resulted in focal synaptic responses, thus proving that NSPC grafts can form a local and spontaneously active synaptic network (Ceto et al., 2020).

Through transcranial optogenetics positioning, a dynamic optogenetics movement map after SCI can be drawn to reflect the damage and recovery of longitudinal structure and function, which expands the traditional applied behavioral and histological analysis and evaluation methods (Qian et al., 2019). Light stimulation of the cuneiform nucleus in Vglut2-ChR2-EYFP mice can induce movement that makes these mice stop abruptly and make sharp turns when approaching a corner. Given that the Vglut2-positive neurons of the cuneiform nucleus are targets for increasing motor activity, it is also likely that they may improve motor function after SCI (van der Zouwen et al., 2021). In fact, optogenetics has recently been used to dissect the spinal cord circuits responsible for evoking rhythmic and stimulating limbs (Llewellyn et al., 2010; Hagglund et al., 2013). Computational model evidence has also shown that optogenetics activate axons abide by the physiological order of diameter from small to large, which is valuable for the recovery of motor function after SCI (Arlow et al., 2013).

### Further Development of Optogenetics Neuromodulation

There are, however, challenges with respect to clinical applications of optogenetics, such as the safe and effective delivery of opsin-encoding vectors and the limitations of transdermal illumination (Mallory et al., 2015). Mondello et al. (2018) have found that different injection methods of adeno-associated virus have a greatly impact on the activation and movement efficiency of the forelimb muscles after illumination, because effective light penetration is limited to the transduced neurons in dorsal layers I through IV of the spinal cord.

It is engineering challenge to be able to make a device that is stretchable and flexible enough to withstand the repeated deformation experienced during normal exercise and to match the low viscoelastic modulus of spinal cord tissue (Chen K. et al., 2017) (see Table 1). The latest progress in chemical materials science has made it possible to develop ultra-miniature LEDs, which can be wirelessly powered and controlled and are suitable for use as implanted devices for optogenetics stimulation of the brain and spinal cord (Canales et al., 2015; Montgomery et al., 2015; Park et al., 2015). Scientists have developed devices based on soft and flexible substrates, including *p*-xylene C (Takeuchi et al., 2005), for electrical stimulation and recording of nerve activity on the surface of the spinal cord of rodents. The implant electronic dura mater that both the mechanical properties that match the statics and dynamics of the host tissue and the bio-integration function that can be implanted for a long time, promote the recovery of motor function in rats after SCI-induced paralysis (Minev et al., 2015). A stretchable transparent electrode array made of a carbon nanotube (CNT) based web-like thin film has been developed that maintains excellent electrochemical performance and broadband optical transparency under stretching. This film also has high durability under cyclic stretching deformation, such that the CNT electrodes can still work during and after rat brain contusion (Zhang et al., 2018). In addition, a fully implantable optoelectronic device that uses near-field wireless communication technology has been developed to allow long-term light stimulation of the spinal cord without restricting the natural behavior of the animals (Montgomery et al., 2015). In transgenic mice, flexible, stretchable probes consisting of thermally drawn polymer fibers coated with micrometer-thick conductive meshes of silver nanowires were used for simultaneous stimulation and recording, which showed the correlation between the electromyogram activity and hindlimb movement caused by light excitation and the local field potential of the spinal cord (Lu et al., 2017).

**Table 1:** Novel electrodes for electrical/optical stimulation and electrical signal recording.

**Electrodes**	**Materials**	**Functions**	**Advantages**	**References**
Carbon-based fiber electrode	Nanostructured carbonaceous materials (graphene and CNT)	Neural stimulation and record long-term solated action potentials and local field potential	High mass-specific surface area, mechanical flexibility, electrical conductivity and biological stability; excellent spatial resolution and selectivity of neural stimulation	Suarez-Perez et al., 2018; Wang et al., 2019; Liu et al., 2020
Flexible electrodes	Polyimide, Titanium, dioxide nanowires	Localized stimulation and detection; resolving high spatiotemporal neural signals	Less invasiveness owing to the ultra-flexible, biocompatibility and stability; excellent electrochemical properties	Tybrandt et al., 2018; Du et al., 2019; Shi and Fang, 2019
Nano-coatings modified electrodes	ZnO nanowires, Pt nanoparticle	Modulating and stimulating neuronal function by delivering the nano-particles; detect electrocorticography signals	Reduce noise, glial encapsulation and decreasing chronic immune response; greatly enhancing the intensity of neural signal detected in vivo; good biocompatibility	Wang et al., 2019; Boehler et al., 2020; Ma et al., 2020
Hydrogel-based electrodes	βVhex and CNTs, Polyvinyl alcohol	Closely mimic the mechanical behavior of neural tissue and safely record biosignals	Greatly reduces the mechanical mismatch at the neural interface, great monitoring signals; perfectly soft, superior biocompatibility and stability	Hong and Lieber, 2019; Oribe et al., 2019; Nam et al., 2020
Micro-LED implant	Two silver plated soft copper core wires with PFA insulation were tightly twisted together	Wirelessly powered and controlled and as implanted devices for optogenetics stimulation of the brain and spinal cord	The implant that causes minimal damage to the spinal cord tissue allows provide optogenetic stimulation in awake, freely moving rats for up to several weeks	Canales et al., 2015; Montgomery et al., 2015; Park et al., 2015; Mondello et al., 2021
Electronic dura mater	A transparent silicone substrate, stretchable gold interconnects, soft electrodes coated with a platinum-silicone composite and a compliant fluidic microchannel	Electrodes transmit electrical excitation and transfer electrophysiological signals	Both the mechanical properties that match the statics and dynamics of the host tissue and the bio-integration function that can be implanted for a long time	Minev et al., 2015
Up-conversion nanoparticle (UCNP)	Yb3+/Er3+/Ca2+-based lanthanide doped up-conversion nanoparticle (UCNP)	Implanting UCNPs in close proximity to related neurons would allow NIR illumination to be converted into visible emission efficiently	Compared to visible light, combine UCNP to NIR illumination offers a higher depth of tissue penetration and less tissue damage	Chen S. et al., 2018; Ao et al., 2019; Ma et al., 2019; Jiang et al., 2020

At present, the traditional stimulus way is to surgically implant the optical fiber to the brain, and implant the optical fiber sleeve near the target area, so that the light activates the neurons in the target area. However, while installing the fiber optic sleeve, the implantation operation will cause a certain degree of invasive damage to the body, which will limit the clinical application of this technology in the future (Adamantidis et al., 2007; Aravanis et al., 2007). How to transmission of light to the deep spinal cord layer of large animals and minimize or eliminate this damage is a challenge that must be overcome to expand the application of optogenetics. The development and application of a wireless implanted optical fiber with a remote control system and near-infrared light (NIR) mediated by up-conversion nanomaterials (UCNPs) provide a promising clinically transformable strategy for nerve repair after SCI (All et al., 2019). Compared to visible light, NIR has stronger penetrating ability, higher sensitivity, lower photobleaching and weaker autofluorescence and causes less photodamage. Research has found that lanthanide (Ln)-doped up-conversion nanoparticles can be used as anti-Stokes shift material to convert low-energy photons into high-energy photons, which have been widely used in the field of biomedicine (Lee and Park, 2018). For example, a Yb^3+^/Er^3+^/Ca^2+^-based lanthanum-doped UCNP effectively converts 808-nm NIR into a green-light wavelength compatible with the cation channel Crimson and can activate a motor circuit in transgenic *C. elegans* (Ao et al., 2019). This approach has also been used successfully in mammals. UCNP was injected into the hippocampus of mice, and the skull was irradiated with NIR. The NIR was then converted into blue light in the hippocampus, which led to the activation of dopaminergic neurons, leading to the release of dopamine from the ventral tegmental area and reducing the incidence of epilepsy (Chen S. et al., 2018). Then, researchers developed ocular injectable photoreceptors combined with UCNPs allowing mice to acquire near-infrared light imaging vision without compromising their normal vision and related behavioral responses infrared vision (Ma et al., 2019). In addition, a UCNP-based multi-effect messenger strategy combined to NIR that triggers the release of nitric oxide in the damaged area on demand has been proposed. UCNPs were constructed for the vector to achieve the recovery of traumatic SCI through simultaneous nerve regeneration and neuroprotection processes, which inhibit glial cell inflammation and promote regeneration (Jiang et al., 2020).

## Combinatorial Approaches

Treatment of SCI presents clinicians with complex and multifaceted obstacles that a single approach is unlikely to overcome. Combination therapy thus is a promising strategy to achieve meaningful functional recovery. Combinational strategies have demonstrated greater beneficial outcomes than their individual components alone by addressing multiple aspects of SCI pathology (Griffin and Bradke, 2020). With the induction of neuronal plasticity and axonal bud burst, rehabilitation training is thought to contribute to the formation of appropriate connections (Hutson and Di Giovanni, 2019). Plasticity-promoting therapies including Chondroitinase ABC (ChABC), which is a bacterial enzyme that degrades chondroitin sulfate, can promote compensatory sprouting of spared fibers to form new neural connections (Garcia-Alias et al., 2008; Griffin and Bradke, 2020). In a rat study, the combination of ChABC and antibodies against NogoA with delayed treadmill rehabilitation training significantly improved axonal germination and functional recovery after partial cervical SCI as compared with a single antibody treatment (Zhao et al., 2013).

Chemical modulation promotes anatomical and functional recovery in subjects with SCI. Compared with electrical modulation regulating local molecular networks, chemical modulation takes effect by targeting specific molecules. Chemical neuromodulation approaches, like the application of compounds and implantable drug pumps, provides strong support for clinical testing in chronic SCI subjects. For example, the co-expression of insulin-like growth factor 1 and osteopontin leads to strong regeneration of CST and recovery of CST-dependent behavior after T10 lateral spinal hemisection (Liu et al., 2017); CPTX, a synthetic synaptic organizer, combining the structural elements of cerebellin-1 and neuron pentraxin-1, promotes restoration of synaptic function and motor coordination in mice of SCI (Suzuki et al., 2020) and KCC2 agonists as promising treatments promote functional recovery after SCI (Chen B. et al., 2018). In addition, a Nogo receptor decoy *via* infusion using osmotic pumps facilitates functional recovery and CST axon growth in non-human primate (Wang et al., 2020). The application of voltage-gated Ca2+ channels inhibitors and neuregulin-1 through implanting of osmotic micro-pump, can also improve the recovery of motor function after SCI in rats (Alizadeh et al., 2018; O'Hare Doig et al., 2020). The combined effect of rehabilitation training, pharmacological regulation and electrical modulation has also been widely verified (Angeli et al., 2018). For example, the combination of serotonin, and dopamine receptor agonists, EES and treadmill training resulted in a remarkable recovery of voluntary motor control in rats following complete transection of the thoracic spinal cord by cross-hemisection (Wenger et al., 2014). EES in combination with intense rehabilitation and neurotransmitter administration helped to restore walking function in three individuals with varying levels of incomplete SCI (Wagner et al., 2018; Griffin and Bradke, 2020). DBS of the midbrain locomotor region and EES of the lumbar spinal cord have been used to tap into the spared circuitry to enable locomotion in individuals with SCI, and when DBS was linked to the intention to walk, this method allowed rats with SCI to carry out volitional walking (Bonizzato et al., 2021). Combination therapy is likely to become the dominant method for SCI gradually.

## Mechanisms of Action

### The Mechanisms of Electrical Stimulation Neuromodulation

SCI has a direct and devastating effect on motor control. The associated breakdown in communication between the brain and spinal cord deprives the intact spinal cord executive center below the injury site of its essential regulation and source of excitement for generating movement (Formento et al., 2018). According to the American Spinal Injury Association, most patients with complete SCI retain some undamaged descending connections, but their spinal cord excitability is severely disrupted, resulting in the loss of function of the intact spinal circuits below the injury (Rossignol and Frigon, 2011; Marder et al., 2014; Eisdorfer et al., 2020). Evidence suggests that neuromodulation technologies can activate the development of sublesional spinal networks, which are isolated from supraspinal commands after SCI, by reestablishing the levels of excitability, and enabling descending motor signals *via* residual connections (Krupa et al., 2020). Whereas the propriospinal networks and the descending reticulospinal commands are putatively the greatest contributors to recovery from anatomically incomplete lesions, recovery from complete lesions is likely due to local lumbar circuit plasticity driven by afferent input (Eisdorfer et al., 2020) (see Figure 2A).

Our understanding of the mechanism of electrical neuromodulation by which sensorimotor function is improved after SCI has remained incomplete. Proprioceptive afferents have been proposed to be the most influential in regaining volitional control of affected muscles (Formento et al., 2018). Ia proprioceptive axons establish direct monosynaptic connections onto motor neurons that innervate agonist muscles. These circuits involving proprioceptive afferents are thought to be critical for locomotor recovery after SCI (Takeoka, 2020). Animal models lacking muscle spindle feedback fail to regain control of affected hindlimbs (Takeoka et al., 2014). However, when EES was applied to the muscle spindle feedback circuit to modulate muscle activity, specific gait, and balance deficits were corrected in rats with incomplete and complete SCI (Moraud et al., 2016). Ablation of proprioceptive afferents severely restricted locomotor recovery and descending circuit reorganization in cases of incomplete SCI (Takeoka and Arber, 2019). Although this procedure works well in animal models, the length of peripheral nerves in humans makes it less effective by increasing the probability of antidromic collisions, thereby reducing the propagation of naturally occurring proprioceptive action potentials (Retamal et al., 2018). A new form of EES using spatiotemporal modulation shows an improvement in human movement because the form does not negatively affect endogenous proprioceptive information (Formento et al., 2018; Wagner et al., 2018). Sensory input provides context-specific information that is critical for motor output generation best suited for any given moment. Behavioral and systems-level evidence strongly indicates that proprioceptive feedback is critical for recovery after a traumatic nervous system injury (Takeoka, 2020).

Propriospinal neurons (PNs) play a crucial role in locomotion by integrating sensory and motor information to coordinate multiple muscle groups (Laliberte et al., 2019; Eisdorfer et al., 2020). The formation and activation of circuits established by spared PNs may promote the re-emergence of locomotion when SCI results in disruption of the flow of motor instructions from the brain and brainstem to the spinal motor circuits (Laliberte et al., 2019). Meanwhile, studies have demonstrated that PNs may be an important CPG supraspinal target for the control of locomotion and forelimb–hindlimb coordination (Ausborn et al., 2019). Although EES does not target PNs directly, there is evidence that EES can indirectly recruit and modulate these circuits by activating peripheral sensory afferent**s**, thereby facilitating hindlimb walking (Moraud et al., 2016; Formento et al., 2018). Indeed, recovery of some volitional control in chronically paralyzed patients may be a consequence of reactivating the circuitry of dormant spared PNs indirectly *via* EES (Angeli et al., 2014). Plasticity among different types of PNs could influence locomotion by enhancing supraspinal drive through relays bypassing the lesion as well as supporting rhythm generation to increase stepping-related patterning (Bui et al., 2016). Supplemental strategies to improve integration of propriospinal interneurons (INs) relays may also prove to be critical for the optimization of locomotor recovery (Krishnan et al., 2019; Laliberte et al., 2019).

There is evidence that sublesional and supralesional spinal circuits can form a translesional spinal network after SCI (Krupa et al., 2020). The existence of this translesional spinal network is one potential mechanism by which electrical stimulation neuromodulation promotes sensorimotor recovery. Evidence largely derived from rodent models of contusion or staggered hemisection suggests that locomotor function depends on relays of spinal INs and connections *via* newly sprouted axons between spared long tracts and propriospinal circuits (May et al., 2017; Krupa et al., 2020). Although substantial progress has been made in understanding the mechanisms underpinning some forms of neuromodulation, much remains to be established.

### The Mechanisms of Optogenetics Neuromodulation

Optogenetics neuromodulation requires selecting and modifying viral vectors that express such transmembrane proteins as Channelrhodopsin (ChR), Halorhodopsin (HR) and Bacteriorhodopsin (BR) in specific populations of neurons. When light of a specific wavelength (390–700 nm) is applied directly to these cells, the conformational changes of these transmembrane proteins cause selective ion current to flow through the cell membrane. An excitatory response can be achieved by activating the ChR2 cation channel with blue light with a wavelength of 470 nm, which allows the influx of positively charged sodium and calcium ions to depolarize neurons (Boyden et al., 2005; Towne et al., 2013). In contrast, inhibition can be achieved by activating a transmembrane chloride and proton pump—either HR or BR, both of which respond to yellow-green light with a wavelength of 580 nm—which leads to the influx of negatively charged chloride ions and hyperpolarizes the neurons (Zhang et al., 2007; Hagglund et al., 2013). Optogenetic stimulation activates functional-dependent neurons, which may alter the plasticity of neurons and restore the neural circuits connection after SCI.

### The Mechanisms of Combinatorial Approaches

There are diverse mechanisms of combinatorial approaches for treating SCI. For example, replacement of damaged cells (cellular transplants) can reduce secondary injury and achieve neuroprotection; providing neuronutritional support (Poplawski et al., 2020), regulating neural activity by neuromodulation and enhancing the intrinsic ability of neuronal regeneration can promote neuronal survival and axon regeneration (Terenzio et al., 2018); targeting myelin-associated inhibitors removes external barriers to axonal regeneration (Silver et al., 2014; Kucher et al., 2018); rehabilitation facilitates the formation of appropriate connections (García-Alías et al., 2009). There are several common combinatorial approaches, such as combining cellular transplants with neurotrophins (Lu et al., 2014), combining cellular transplants with anti-inhibitory therapies (DePaul et al., 2017), combining anti-inhibitory therapies with neurotrophins (Elliott Donaghue et al., 2016), combination treatments involving targeting the intrinsic growth response (Wang et al., 2018), combinations of neuromodulation with rehabilitation (Chen K. et al., 2017) and so on. Combinational strategies have demonstrated greater beneficial outcomes than their individual components alone by addressing multiple aspects of SCI pathology, resulting in clinically relevant functional improvements (Christiansen and Perez, 2018; Bonizzato et al., 2021). The mechanisms of combinatorial approaches for treating SCI are complex rather than simple overlay of multiple kinds of mechanisms. Combining optogenetics neuromodulation with electrical neuromodulation, and combination cellular transplants with neuromodulation both need to be further explored, which may bring new possibilities for the treatment of SCI.

## Discussion

Neuromodulation for the treatment of SCI is a rapidly developing field with great potential. With the expansion of clinical trials and increasing clinical and experimental evidence, neuromodulation-based interventions are more likely to lead to recovery of motor and sensory function (James et al., 2018). However, neuromodulation for SCI is still in its infancy.

### Challenges for Electrical Neuromodulation Applications

#### Limitation and Development of Neuromodulation Devices

The widespread and long-term use of neuromodulation devices in the clinic is currently limited by a number of factors, including price, availability, and the expertise required to operate certain devices. Although implantable nerve electrodes have strong advantages, the durability of implanted electrodes is a common problem. Implanted electrodes can cause tissue damage and glial growth around the implant site, resulting in inefficient recording, and stimulation (Lee et al., 2016). In contrast, tcSCS and tDCS have the potential to be rapidly and widely delivered to patients with SCI (James et al., 2018). Therefore, some researchers believe that tcSCS and tDCS are safer and more accessible treatment methods for some patients with SCI (Taccola et al., 2020).

How to achieve long-term biosignal recording is a prominent challenge across the entire field of bioelectronic medicine (Lee et al., 2016; Bouton, 2019). Recent advances in the optimization of neural electrode–tissue interfaces—including electrode materials (based on graphene or CNT fibers), electrode structures (flexible electrodes), nano-coatings and hydrogel-based neural interfaces—have helped to achieve this goal (Liu et al., 2020) (see Table 1). These optimization methods can effectively improve the long-term stability of the neural interface and chronic inflammatory response during the process of biosignal recording and regulation. Another category of devices, wearable electronic devices, could fully address the problems of implanted electrodes and make it possible to monitor physiological signals in real time (Bouton, 2019), although the effectiveness and accuracy of such devices need to be further developed.

#### Development of a Delivery System for Electrode Implantation

To optimize electrode design and targeting, another major challenge is the development of an appropriate delivery system for electrode implantation. The use of stereotactic frames or frameless positioning systems during spinal surgery can lead to problems of inaccuracy due to the variability of the surface markers used (Lobel and Lee, 2014). Linking multimodal *in vivo* neuroimaging with neuromodulation strategies is a powerful combination that is expected to significantly advance neuromodulation technologies and provide more precise and effective treatment for refractory neurologic disorders (Edwards et al., 2017). Current advances in real-time magnetic resonance imaging coupled with frameless stereotactic approaches are enabling faster, more precise placement of DBS electrodes (Starr et al., 2010; Edwards et al., 2017).

#### Problems to Be Solved in Clinical Trials of Neuromodulation

The selection of participants and the determination of key stimulus parameters and timing of intervention are important considerations in the design of neuromodulation intervention trials for SCI. The design of the trial needs to consider whether neuromodulation intervention should be used in the early (plastic) or late (stable) stages of SCI, especially when combined with rehabilitation training (James et al., 2018). The influence of different stimulation sites and parameters on treatment outcomes is significant but is typically not addressed. Increased understanding of the mechanisms of neuromodulation would be of great help in solving these problems. It should be noted that electrical neuromodulation is a very complex technology. Except for clinical trials, patients should not rush into electrical stimulation therapy, lest more serious consequences occur (Willyard, 2019).

#### Understanding the Mechanisms of Neuromodulation

Despite the rapid development of neuromodulation as a treatment for SCI, we do not know much about its underlying mechanisms. In fact, this severely restricts the further development and wide application of neuromodulation. Are there certain coding rules for the electrical signal that can effectively improve the plasticity of the central nervous system? What are the effective stimulus parameters? Do the different parameters have a relatively fixed range of therapeutic effects or do they change in real time? When do stimulatory interventions have the best effect on sensorimotor recovery? Which gene expression changes in the molecular network are controlled by neural regulation? The answers to these questions will greatly promote the application of neuromodulation in clinical trials for SCI.

### Challenges for Optogenetics Neuromodulation Applications

The further development of optogenetics technology is expected to overcome some limitations of electrical stimulation in reshaping spinal circuit and restoring motor function after SCI. Firstly, optogenetics neuromodulation allows to selective neurons activation and fine loop control in spinal cord, as the specificity of lentiviral transfection of selected motor neurons increases (Jackman et al., 2018). Secondly, optogenetics may restore function in a more physiologically relevant way, particularly for functions that involve complex patterns of excitation and inhibition among different subpopulation of neurons (Vila et al., 2019). Despite the significant advantages of optogenetics neuromodulation over electrical stimulation, several limitations must be addressed before optogenetics can be used clinically to restore function in SCI patients. Firstly, it is necessary to development and design more safe and efficient gene delivery vectors to target spinal cord tissue. Secondly, it is critical to extend more light-sensitive proteins in the wavelength range of red light to NIR to maintain low-dose light to maximize light penetration and reduce light damage (Shen et al., 2020). Thirdly, stimulation systems need to be developed to optimize light transmission patterns in a tissue-specific manner while reducing glial responses to light transmission devices. Although challenges remain, there is substantial evidence that neuromodulation is an effective treatment for SCI. With advances in neuromodulation devices, experimental techniques and computer power, as well as a growing understanding of the mechanisms involved, neuromodulation is rapidly becoming an important modality for the recovery of meaningful function after SCI.

## Author Contributions

HZ and Yaping Liu wrote the review. KZ and WW prepared the figures. Yaobo Liu provided supervision. All authors contributed to the article and approved the submitted version.

## Funding

Our study was financially supported by the National Natural Sciences Foundation of China (numbers 82171376, 81971164, 81330026, 81771330), the National Key Basic Research Development Program of the Ministry of Science and Technology of China (973 Program, 2013CB945600), a project funded by the Priority Academic Program Development of Jiangsu Higher Education Institutions, and the Key Research and Development Plan of Jiangsu Province (BE2018654).

## Conflict of Interest

The authors declare that the research was conducted in the absence of any commercial or financial relationships that could be construed as a potential conflict of interest.

## Publisher's Note

All claims expressed in this article are solely those of the authors and do not necessarily represent those of their affiliated organizations, or those of the publisher, the editors and the reviewers. Any product that may be evaluated in this article, or claim that may be made by its manufacturer, is not guaranteed or endorsed by the publisher.
